# Sacral nerve stimulation prompts vagally‐mediated amelioration of rodent colitis

**DOI:** 10.14814/phy2.14294

**Published:** 2020-01-10

**Authors:** Trisha S. Pasricha, Han Zhang, Nina Zhang, Jiande D. Z. Chen

**Affiliations:** ^1^ Department of Medicine Johns Hopkins Hospital Baltimore MD USA; ^2^ Division of Gastroenterology and Hepatology Johns Hopkins School of Medicine Baltimore MD USA

**Keywords:** DSS colitis, heart rate variability, inflammatory bowel disease, sacral nerve stimulation, sympathovagal balance

## Abstract

Neuromodulation based on the vagal anti‐inflammatory reflex has emerged as an exciting therapeutic approach for chronic inflammatory diseases. However, it is unclear whether direct stimulation of the vagus or of pelvic nerves coming from sacral roots, providing the bulk of colonic parasympathetic innervation, is the best approach. We hypothesized that sacral nerve stimulation (SNS) would be an effective treatment for colitis. Age and sex‐matched Sprague‐Dawley rats were administered 5% dextran sulphate sodium (DSS) in drinking water ad libitum for 7 days. A group of rats was sacrificed after DSS treatment, and the remaining rats were randomized to either sham‐SNS or SNS groups, which were performed for 1 hr daily for 10 days. Stimulations were delivered via chronically implanted electrodes using an 8‐channel universal pulse generator. Sacral nerve stimulation promoted recovery of colitis demonstrated by decreased disease activity index, myeloperoxidase activity, tissue TNF‐alpha, and histological scores as well as an increased colonic M2 macrophage population. Heart rate variability analysis demonstrated a decrease in low frequency and increase in high frequency with SNS, corresponding to increased vagal tone. Additionally, plasma pancreatic peptide was increased and norepinephrine was decreased after SNS in colitis while colon tissue acetylcholine was increased with SNS. This is the first study to the best of our knowledge that demonstrates the benefit of SNS with autonomic mediation. SNS alters the expression of inflammatory cytokines and macrophages as well as modulates neurotransmitters involved in systemic inflammation.

## Graphical abstract

Sacral nerve stimulation has shown clinical promise in other GI conditions such as fecal incontinence; however, the mechanism of sacral nerve stimulation has not been fully elucidated. In a DSS rodent model, we show that sacral nerve stimulation ameliorates colitis, and is associated with increased vagal expression. These results indicate that sacral nerve stimulation may signal via an autonomic mechanism to potentially attenuate inflammatory bowel disease in humans.

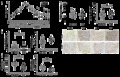

## 1 INTRODUCTION

Neuromodulation based on the cholinergic anti‐inflammatory pathway has emerged as an exciting therapeutic approach for the treatment of a wide variety of diseases including rheumatoid arthritis, sepsis, chronic pain, seizure disorders, and obesity (Johnson & Wilson, 2018). The bidirectional brain‐gut axis is mediated via the autonomic nervous system, by which the vagus nerve functions via both afferent and efferent anti‐inflammatory pathways. Parasympathetic activity enhanced by inflammatory conditions leads to acetylcholine release, and this has been shown to dampen inflammatory cytokine production by macrophages (Bonaz et al. (2016)). Stimulation of the vagus nerve has resulted in promising experimental evidence in an early 6‐month pilot of Crohn's disease patients (Borovikova et al., 2000).

However, it is not clear whether direct stimulation of the vagus nerve or the pelvic nerves coming from sacral roots, providing the bulk of parasympathetic innervation to the colon, is the best approach for attenuating colonic inflammation. Compared to the vagus nerve, the sacral nerve affords the ease of minimally invasive surgical access that can be performed under local anesthesia in the outpatient setting, and is already currently approved for human use in urgency urinary frequency and fecal incontinence, for which it is a mainstay of treatment (Noblett and Buono, (2018)). A study of SNS in a porcine TNBS‐colitis model demonstrated enhanced morphological and functional recovery of the intestinal epithelial barrier (Bregeon et al., 2016). We recently showed that SNS is as effective as VNS in attenuating TNBS‐colitis in a rat model (Guo et al., 2019). Whether the potential benefits of SNS in IBD involve a global autonomic mechanism remains unclear.

Here we sought to determine the effect of sacral nerve stimulation in a rodent dextran sulfate sodium (DSS) model of colitis and assess the role, if any, of autonomic function.

## 2 METHODS

### 2.1 Experimental model

Male Sprague‐Dawley (*SD*) rats (8 weeks old; 250–275 g; Envigo International holdings Inc.) were housed at the Johns Hopkins Animal Facility and fed a standard diet without phytogenic feed additives and maintained under the following conditions: Temperature between 20 and 22°C, relative humidity between 50% and 60%, and 12/12‐hr light/dark cycle. Rats were allowed to acclimate to these conditions for at least 7 days before experimentation. Care and experimentation of rats were performed in accordance with institutional guidelines under protocols approved by the Institutional Animal Care and Use Committee at the Johns Hopkins University.

Colitis was induced using dextran sulfate sodium (DSS). Rats were administrated 5% DSS (molecular weight 40 kD; Affymetrix USB, Santa Clara, CA) in drinking water ad libitum for 7 days (*n* = 6–8 in each group). Control rats received the same drinking water without DSS (*n* = 6).

### 2.2 Sacral nerve stimulation and sham sacral nerve stimulation therapies

At day 7 following induction with DSS, animals were randomized assigned into the following three groups: (a) Colitis group (*n* = 6): rats were sacrificed; (b) Sham‐SNS group (*n* = 8): rats with electrode placement, but no stimulation for 10 days and (c) SNS group (*n* = 8): rats with electrode placement and stimulation for 10 days. The duration of treatments were chosen based on preliminary tests in our laboratory. DSS at the dose used in this study for longer than seven days resulted in too severe inflammation and SNS for longer than 10 days was complicated by spontaneous recovery of inflammation.

Animals were operated under anesthesia induced by isoflurane (Abbott Laboratories). For the SNS group, a pair of electrodes (Streamline 6494, Medtronic) was placed around right sacral nerve (S3), and the exposed wire was isolated from circumjacent tissues using dental cement. The SNS electrode connecting wires were fixed in the muscle layer near the implant and brought out via a tunnel underneath the skin. The connector was linked to a universal pulse generator (Model DS8000, World Precision Instruments). Animals in the Sham‐SNS group underwent the same surgical procedures for the placement of electrodes, except no electrical stimulation was delivered during the treatment.

Sacral nerve stimulation was applied for 1 hr daily for a period of 10 consecutive days after colitis was induced. The parameters were as follows: 5 Hz, 0.5 ms, 10 sec on, 90 sec off, 90%MT (motor threshold). Stimulations were delivered via the chronically implanted SNS electrodes using an 8‐channel universal pulse generator (Model DS8000, World Precision 158 Instruments).

At the end of study, rats were sacrificed by CO_2_ euthanasia, rapidly dissected, and colon lengths were measured. The entire colon was quickly removed and gently cleared of feces. Small segments from proximal and distal colon, respectively, were fixed in 10% buffered formalin for 24 hr at room temperature before embedded in paraffin for histology analysis. Then the mucosa was scraped from the rest of colon at 4°C, and samples were snap‐frozen, and stored at −80°C until further analysis. Blood was collected from tail vein in endotoxin‐free silicone coated tubes with/without EDTA before DSS treatment (Day 0), after induction of colitis with DSS (Day 7) and after 10 days of stimulation (Day 17). The blood samples were centrifugation (2,200*g*, 4°C, 10 min) and the plasma was collected and stored at −80°C until analyzed.

**Figure 1:**
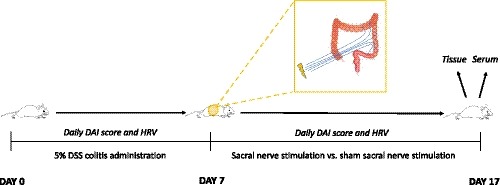
Study timeline. Rats were treated for 7 days with 5% DSS during which daily DAI scores and HRV were measured. After this, rats were either treated with sacral nerve stimulation or sham‐sacral nerve stimulation for an additional 10 days. Measurements of DAI and HRV continued during this time period. At the end of the study on day 17, rats were sacrificed and tissue and serum were collected for analysis

Our study design is summarized in Figure 1.

### 2.3 Disease activity index

Daily disease activity index (DAI) scores were assessed based on following parameters: weight loss, stool consistency, and bleeding using a previously published system (Jin et al., 2017). Each item was rated from 0 to 4, yielding a total of score from 0 (healthy) to 12 (maximum colitis severity).

### 2.4 Heart rate variability

The autonomic function was assessed by the spectral analysis of the heart rate variability (HRV). The HRV signal was derived from the original ECG recording by identifying R waves, interpolating R‐R interval data (bilinear interpolation) at a range of 1.5 Hz to 100 Hz, and, finally, downsampling the interpolated HRV data at 8 Hz suitable for spectral analysis. Spectral analysis of the HRV signal was performed using the smoothed spectral analysis method. Spectral powers at two frequency ranges calculated: (a) a high‐frequency band (HF; 0.8 to 4.0 Hz) reflecting cardiac vagal efferent activity; and (b) a low‐frequency band (LF; 0.3 to 0.8 Hz) reflecting mainly sympathetic activity.

ECG was recorded on experimental Day 0 (30‐min recording as baseline), Day 7 (after induction of colitis), and Day 18 (30‐min recordings after SNS or sham‐SNS).

### 2.5 MPO, cytokine, and neurotransmitter analysis

Neutrophil influx in tissue was analyzed by assaying the enzymatic activity of MPO, a marker for neutrophils. The MPO activity of colonic mucosa samples was assessed using an MPO colorimetric activity assay kit (Sigma‐Aldrich) according to the manual.

Colon tissue cytokines were analyzed utilizing a multiplex sandwich immunoassay from MILLIPLEX MAP kit (EMD Millipore) containing fluorescence‐labeled microspheres conjugated with monoclonal antibodies to a variety of target cytokines.

Plasma from different time points in different groups were tested for PP and NE in compliance with the instructions of ELISA kit (Ebioscience Company). Tissue lysates were used for testing Ach, iNOS, VIP, and substance P, which were determined using commercially available immunoassay kits according to the manufacturer's instructions. In summary, the diluted samples (plasma or tissue lysates) were placed in a 96‐well plate incubated with precoated anti‐rat IgG antibody, washed and developed, and quantified. Inter‐assay variation was avoided by assaying all the samples on the same day.

### 2.6 Histology

Rat colons were fixed in 10% buffered formalin for 24 hr at room temperature before embedded in paraffin. Tissues were then sectioned at 4‐μm thickness and stained with hematoxylin & eosin (H&E) using standard protocols. H&E stained slides were scored by two pathologists. Each colon was assigned four scores based on the degree of epithelial damage and inflammatory infiltrate in the mucosa, submucosa, and muscularis/serosa. The following histological parameters were obtained: for inflammatory infiltration, grading was considered as severe = 3, moderate = 2, mild = 1, absent = 0; for hyperplasia, grading was considered as severe = 3, moderate = 2, mild = 1, absent = 0; for ulceration, grading was considered as diffuse glandular disruption or extensive deep ulceration = 4, glandular disruption or focal deep ulceration = 3, diffuse superficial ulceration = 2, focal superficial ulceration = 1, absent = 0. The three sub‐scores were summed as the total colitis inflammatory index. The scores for each of the 6–8 rats per treatment group were averaged.

### 2.7 Macrophage analysis

Slides were stained with CD68 (pan macrophage stain) and CD206 (selective M2 macrophage stain). Total macrophage and M2 macrophages were counted in representative slides from each study group by blinded reviewers. These were then averaged and the number of macrophages reported.

### 2.8 Statistical analyses and manuscript preparation

One‐way analysis of variance (ANOVA) and *t*‐test were used to determine the differences among the groups with the aid of GRAPHPAD PRISM 8 software (GraphPad Software). Individual data points were plotted with superimposed mean—standard deviation. Correlation analysis were investigated using the Pearson correlation coefficients by SPSS software (Version 22.0, IBM Corporation). Values of *p* < .05 were considered statistically significant. All authors had access to the study data and reviewed and approved the final manuscript.

## 3 RESULTS

**Figure 2:**
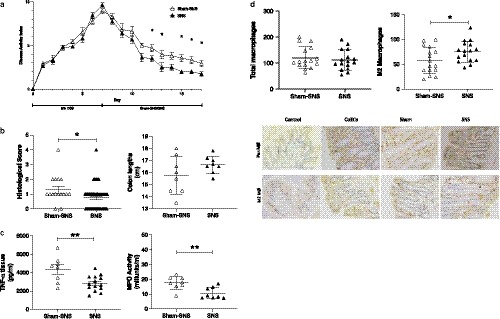
Sacral nerve stimulation promotes recovery of colitis. (a) DAI is significantly improved with SNS within 5 days of treatment. (b) Overall histological scores are significantly improved and colon lengths trend toward improvement with SNS. (c) Tissue TNF‐alpha and MPO activity, a marker of inflammation, are significantly decreased with SNS. (d) Macrophage population and representative colon biopsies demonstrating increased M2 macrophages after SNS (H&E with CD68 and CD206 staining, 100×). Mean values with standard deviation are shown. * denotes *p* < .05

Induction of colitis with DSS resulted in significant clinical and histological deterioration as well as in increased inflammatory cytokines. We observed that SNS promoted clinical improvement within five days of treatment, ameliorating diarrhea, bleeding, and weight loss, evidenced by improved DAI score versus sham‐SNS, all *p* < .05, Figure 2a. Microscopically, oral administration of DSS induced a mild inflammation characterized by diffusion of granulocytes, leukomonocytes, inflammatory infiltrates, ulcerations, and goblet cell depletion. After ten days of SNS, there was a reduction in colonic damage, including the inhabitation of inflammatory infiltration, ulcer healing, the presence of goblet cells in the mucosal layer, and a progressive restoration of the colonic architecture. Overall histological scores were significantly attenuated by SNS (versus sham‐SNS, *p* < .05), and there was a trend toward improvement in colonic length (versus sham‐SNS, *p* = .06, Figure 2b).). Our data also showed that SNS significantly reduced MPO activity in colonic tissue by 57% (versus sham‐SNS, *p* < .05), and substantially decreased the colonic tissue TNF‐α (versus sham‐SNS, all *p* < .05, Figure 2c). Interestingly, while there was no difference in the total number of macrophages between SNS and sham‐SNS, the SNS group had a significant increase in the M2 macrophage population (versus sham‐SNS, *p* < .05, Figure 2d).

**Figure 3:**
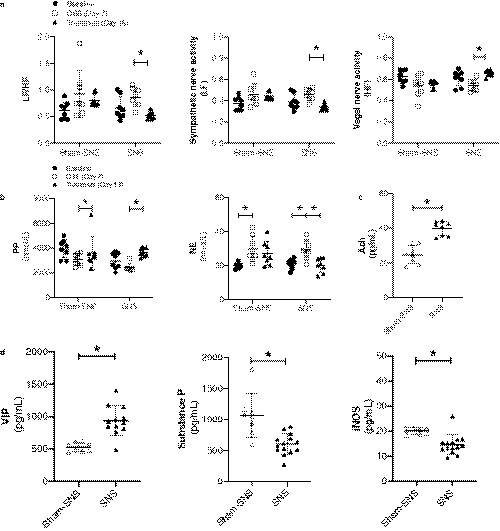
Sacral nerve stimulation is associated with increased parasympathetic activity. (a) LF/HF ratio is significantly reduced after SNS in colitis with a decrease in LF and increase in HF. (b) Plasma PP is increased and NE is decreased after SNS in colitis. (c) Colon tissue Ach is significantly increased with SNS. (d) Plasma VIP, iNOS, and substance *p* are significantly regulated by SNS. Mean values with standard deviation are shown. * denotes *p* < .05

Next we evaluated the role of autonomic function and corresponding sympathovagal neurotransmitters in our model. First, we studied the effects of SNS on spectral analysis of heart rate variability (HRV) (Figure 3a). Interestingly, we showed that SNS substantially increased vagal activity and decreased sympathetic activity after induction of colitis (vs. DSS alone, all *p* < .05). We also demonstrated that while DSS trended toward decreased plasma pancreatic polypeptide and increased norepinephrine, this was significantly reversed by SNS (vs. DSS alone, and vs. sham‐SNS, all *p* < .05, Figure 3b). Furthermore, colonic tissue acetylcholine (ACh) levels were significantly elevated by SNS (vs. Sham‐SNS, *p* < .05, Figure 3c).

We then sought to further clarify the relationship between sympathovagal balance and these neurotransmitters (Table 1). Our data showed that tissue Ach level was positively correlated to HF, representing vagal activity (*R* = .439, *p* = .022), and negatively correlated to LF, representing sympathetic activity (*R* = −.439, *p* = .022). However, HRV did not exhibit significant correlation with PP/NE.

**Table 1:** Correlations among Ach, PP, NE, VIP, Substance P, and iNOs with HF, LF, and LF/HF ratio

		HF	LF	LF/HF
Ach	Pearson correlation	.439*	−.439*	−.435*
	Sig. (two‐tailed)	.022	.022	.023
	*N*	27	27	27
PP	Pearson correlation	.132	−.132	−.138
	Sig. (two‐tailed)	.510	.510	.494
	*N*	27	27	27
NE	Pearson correlation	−.030	.030	.315
	Sig. (two‐tailed)	.092	.092	.109
	*N*	27	27	27
VIP	Pearson correlation	.670*	−.670*	−.613*
	Sig. (two‐tailed)	.001	.001	.001
	*N*	21	21	21
Substance P	Pearson correlation	−.445*	.445*	.431
	Sig. (two‐tailed)	.043	.043	.051
	*N*	21	21	21
iNOS	Pearson correlation	−.608*	.608*	.626*
	Sig. (two‐tailed)	.003	.003	.002
	*N*	21	21	21

* *p*<.05.

Plasma inflammomodulatory neurotransmitters were studied next (Figure 3d). The pro‐inflammatory substance P was dramatically elevated by DSS administration, and SNS significantly decreased substance P level as compared to Sham‐SNS (*p* < .05). The anti‐inflammatory vasoactive intestinal peptide (VIP) level was significantly higher in SNS group than that in Sham‐SNS group, although DSS alone did not have a dramatic impact on VIP level (vs. control, *p* > .05). Pro‐inflammatory inducible nitric oxide synthetase (iNOS) was mildly provoked by DSS and decreased much more upon SNS (vs. sham‐SNS, *p* = <.05). All three neurotransmitters showed strong correlation with autonomic function presented by HRV (Table 1). VIP was positively related to vagal activity (*R* = .633, *p* < .05) while substance P and iNOs were negatively related to vagal activity (R = −.445, *p* < .05 and *R* = −.608, *p* < .05, respectively).

## 4 DISCUSSION

To the best of our knowledge, prior studies have demonstrated the benefits of VNS (but not SNS) for gut inflammation in animal models; however, this is the first study to demonstrate the benefit of SNS involving autonomic mediation. Our data indicate that SNS can ameliorate colitis both clinically as shown by our DAI data and histologically. We demonstrate that SNS alters the expression of inflammatory cytokines and boosts anti‐inflammatory M2 macrophages in colitis, underscoring the translational benefit of this technique in future for human inflammatory bowel disease.

Our second aim was to investigate any role of global autonomic function in SNS. Our data suggest that SNS modulates neurotransmitters involved in systemic inflammation. Heart rate variability is a well‐recognized metric of vagal tone, and studies in human IBD have shown a correlation between decreased parasympathetic activity measured via HRV and increased inflammatory markers (Engel, Ben‐Horin, & Beer‐Gabel, 2015; Gunterberg, Simren, & Ohman, 2016). In our study, we establish that SNS alters the sympathovagal balance evidenced by changes in HRV and neurotransmitters, with increased Ach and PP (as markers of parasympathetic activity) and decreased NE (as a marker of sympathetic activity) seen in SNS. We furthermore demonstrate a link between these and other inflammomodulatory neurotransmitters with HRV, indicative of a vagally‐mediated response.

Recent studies have upended long‐held dogma with convincing evidence that sacral nerves are, in fact, sympathetic in terms of ontogeny and molecular signatures, and that there may be no spinal counterpart of the cranial parasympathetic system (Espinosa‐Medina et al., 2016). Functionally, however, our HRV data suggest an association in SNS with increased systemic parasympathetic activity, although it remains unclear whether and to what extent SNS directly activates local pelvic splanchnic nerves in the colon versus a vagal reflex arc, potentially involving the cholinergic anti‐inflammatory pathway. Additional studies are required to fully elucidate the exact anti‐inflammatory signaling pathway of SNS, including investigation of the effect of SNS on motility and secretion.

In summary, we have shown that SNS promotes vagally‐mediated recovery in a rodent model of colitis. Our data suggests that SNS is a promising and novel therapeutic option for colitis and perhaps other forms of IBD.

## CONFLICT OF INTEREST

The authors have no conflicts of interest to declare.

## AUTHOR CONTRIBUTIONS

Pasricha analyzed and interpreted data and drafted the manuscript. Zhang H and Zhang N performed experiments and analyzed data, Chen designed experiments, analyzed, and interpreted data and performed critical revision of the manuscript. Each author approved the final draft submitted.
